# Isoprene Production on Enzymatic Hydrolysate of Peanut Hull Using Different Pretreatment Methods

**DOI:** 10.1155/2016/4342892

**Published:** 2016-10-25

**Authors:** Sumeng Wang, Ruichao Li, Xiaohua Yi, Tigao Fang, Jianming Yang, Hyeun-Jong Bae

**Affiliations:** ^1^Key Lab of Plant Biotechnology in Universities of Shandong Province, College of Life Sciences, Qingdao Agricultural University, Qingdao 266109, China; ^2^Bio-Energy Research Institute, Chonnam National University, Gwangju 500-757, Republic of Korea

## Abstract

The present study is about the use of peanut hull for isoprene production. In this study, two pretreatment methods, hydrogen peroxide-acetic acid (HPAC) and popping, were employed prior to enzymatic hydrolysis, which could destroy the lignocellulosic structure and accordingly improve the efficiency of enzymatic hydrolysis. It is proven that the isoprene production on enzymatic hydrolysate with HPAC pretreatment is about 1.9-fold higher than that of popping pretreatment. Moreover, through High Performance Liquid Chromatography (HPLC) analysis, the amount and category of inhibitors such as formic acid, acetic acid, and HMF were assayed and were varied in different enzymatic hydrolysates, which may be the reason leading to a decrease in isoprene production during fermentation. To further increase the isoprene yield, the enzymatic hydrolysate of HPAC was detoxified by activated carbon. As a result, using the detoxified enzymatic hydrolysate as the carbon source, the engineered strain YJM21 could accumulate 297.5 mg/L isoprene, which accounted for about 90% of isoprene production by YJM21 fermented on pure glucose (338.6 mg/L). This work is thought to be the first attempt on isoprene production by* E. coli* using peanut hull as the feedstock. More importantly, it also shows the prospect of peanut hull to be considered as an alternative feedstock for bio-based chemicals or biofuels production due to its easy access and high polysaccharide content.

## 1. Introduction

Isoprene (2-methylbuta-1,3-diene), as a polymer building block, plays a pertinent role in the synthetic chemistry industry and represents an important biological material. Isoprene could serve as the feedstock not only in industrial production of synthetic rubber or aviation fuel [1, 2] but also in the fields of isoprenoid medicines and fragrances [3]. Currently, industrial isoprene production mainly relies on fossil sources, achieved by means of chemical synthesis techniques [1, 4]. However, due to the decrease of petroleum reserve and the enhancement of environmental awareness, it becomes increasingly urgent and necessary to produce isoprene using renewable resources as an alternative to petroleum resource.

Although isoprene could be produced from many kinds of plants [5] or some microorganisms such as fungi,* Eurotium amstelodami* [6], both ways still sound impractical, since it is difficult to harvest isoprene from plant species [7], and the material shortage and low conversion efficiency are widely recognized as a bottleneck for isoprene production by microorganisms.

**Figure 1:**
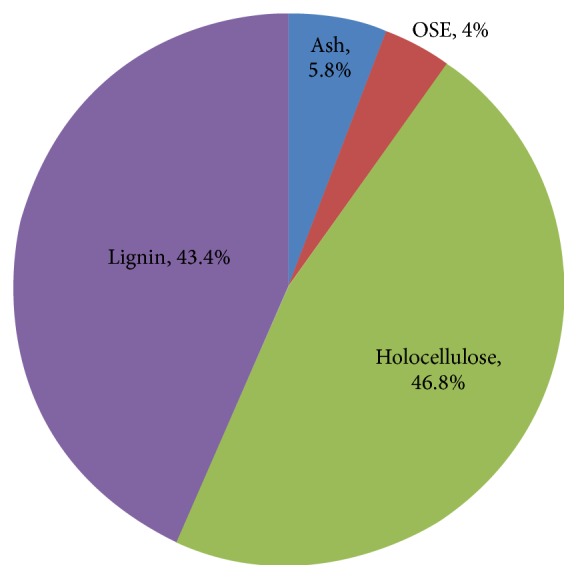
Chemical composition of peanut hull.

Today, millions of tons of agricultural lignocellulosic wastes are produced around the world annually. The abundant supply and low cost properties [8, 9] have made agricultural lignocellulosic wastes the most promising materials for substituting the dwindling fossil fuels. In China, the annual production of peanut could reach up to 1.3 × 10^7^ tons, which accordingly resulted in 3.64 × 10^6^ tons of peanut hull in 2008 [10]. Recently, the USDA reported that peanut production in China accounted for approximately 45% of the total yield of the world's peanut [11] (USDA 2015). As is shown in Figure 1, peanut hull consists of 46.8% holocellulose, 5.8% ash, 4.0% OSE, and 43.4% Klason lignin. Its high polysaccharide content makes peanut hull a suitable feedstock for the production of bio-based chemicals or biofuels including isoprene.

Since cellulose is usually surrounded by hemicellulose and lignin which would reduce the conversion rate of cellulose into fermentable sugar, it is vital to develop an economic pretreatment method to change the lignocellulosic biomass structure so as to improve degradation efficiency by cellulase to translate cellulose into fermentative saccharides. So far, various pretreatment techniques have been developed to disrupt the lignocellulosic structure prior to enzyme hydrolysis, including dilute acid, steam explosion, liquid hot water, ammonia pretreatments, and popping [12, 13]. Among them, the machine used in popping pretreatment is a very simple system consisting of direct burner and rotary reactor without steam generator [12], and this process has remarkable advantages including higher saccharification efficiency, cost effectiveness, and environmental safety [14]. Acetic acid might enhance the hydrolysis efficiency of hemicellulose [15], and hydrogen peroxide pretreatment has many advantages such as forming fewer inhibitors and generating more glucose yield in addition to lower toxicity and less environmental impact [15, 16].

Based on the above analysis, in this study, we introduced two pretreatment methods to treat peanut hull prior to hydrolysis by cellulase, popping [17] and HPAC [18], with HPAC being an efficient method, including hydrogen peroxide and acetic acid. Meanwhile, to further enhance the isoprene production, we detoxified the enzymatic hydrolysate of peanut hull pretreated by HPAC. We finally achieved the different isoprene production with several kinds of enzymatic hydrolysates. This work is the first attempt to produce isoprene by* E. coli* using the peanut hull as the feedstock. And, more importantly, this work provides evidence to show that another lignocellulose material, peanut hull, could be considered as a promising feedstock for industrial production of bio-based chemicals or biofuels.

## 2. Materials and Methods

### 2.1. HPAC Pretreatment and Popping Pretreatment

Peanut hull (PH) used in this experiment was collected from Shandong province, China. PH was milled and screened to a 40–60 mesh size and then was air-dried after its associated wastes were washed by running water. To be specific, ten-gram peanut hulls (PHs) were treated with 100 mL of HPAC solution, a mixture of hydrogen peroxide and acetic acid (1 : 1; v/v), and then incubated at 80°C for 3 h, after which materials were filtered to separate the HPAC solution from the solid residue, and they were washed 3 times by running water until neutral pH was reached [18].

A total of 100 g (dry weight) of PHs was treated using the popping equipment [17]. Filled with PHs (moisture content: 75%), the reactor was directly heated with a gas burner at a rate between 15 and 20°C/min and rapidly opened the hatch at 220°C and 1.47 MPa. After treatment, materials were recovered in a reactor and cooled at room temperature. And then HPAC and popping treated PHs were dried by a lyophilizer at −45°C for 5 days.

### 2.2. SEM Imaging

The surface morphologies of samples, including pretreated PHs by popping and HPAC and untreated PH, were analyzed using scanning election microscopy (SEM; JSM-7500F, Jeol, Japan). Imaging was captured at a beam voltage of 4 kV. Prior to observation and photography, biomass samples were dried at 50°C for 24 h and gold sputter-coated (20 nm).

### 2.3. Chemical Composition Analysis

Both 20 mg raw and pretreated PHs were used to analyze chemical composition. The structural carbohydrates, ash, and lignin analysis procedure of all biomass samples were measured according to the NREL Laboratory Analytical Procedure (LAP) [19]. And organic solvent extractives (OSE) were analyzed with TAPPI Standard Methods [20]. The raw and pretreated (HPAC and popping) PHs were analyzed for their neutral sugar content using gas chromatography (GC) [13]. The samples were analyzed via GC (GC-2010; Shimadzu, Otsu, Japan) using a DB-225 capillary column (30 m × 0.25 mm i.d., 0.25 *μ*m film thickness, J&W; Agilent, Folsom, CA, USA) operated with helium. The operating conditions were as follows: injector temperature of 220°C, flame ionization detector (FID) at 250°C, and an oven temperature of 100°C for 1.5 min with a constant increase of 5°C/min to 220°C.

### 2.4. Enzymatic Hydrolysis

The PHs as 1% (w/v) substrate were treated in 50 mM sodium citrate buffer (pH 4.8) supplemented with 0.01% (w/v) sodium azide. Each of the enzymes, celluclast (Novozymes, Denmark) and xylanase (endo-1,4-*β*-xylanase from* Trichoderma longibrachiatum*, Sigma-Aldrich, USA), were loaded with 30 FPU per gram of glucan and 300 international units (IU)/mL, respectively. All samples were completely suspended in rotary shaker at 200 rpm at 37°C for 48 h. All enzymatic hydrolysis experiments were performed in triplicate.

### 2.5. Detoxification with Activated Carbon

The enzymatic hydrolysate of peanut hull (HPAC pretreated) along with 1% (w/v) activated carbon (05-690A, 50–200 mesh, Fisher Scientific Co., Pittsburgh, PA, USA) was mixed in 250 mL flask. And then the flask had been incubating at 30°C with shaking at the rate of 180 rpm for 10 h. After treatment, the activated carbon was removed from the mixture by centrifugation at 10000 rpm for 10 min. To get the detoxification hydrolysate, the supernate was finally filtered by using 0.2 *μ*m filter membrane.

### 2.6. Shake Flask Fermentation

Shake flask experiments were carried out in triplicate using a series of 25 mL sealed shake flasks containing 5 mL fermentation medium including glucose 2 g/L or suitable concentration of enzymatic hydrolysate, K_2_HPO_4_ 9.8 g/L, beef extract 9 g/L, ferric ammonium citrate 0.3 g/L, citric acid monohydrate 2.1 g/L, MgSO_4_ 0.06 g/L, and 1 mL trace element solution, consisting of (NH_4_)_6_Mo_7_O_24_·4H_2_O 0.37 g/L, ZnSO_4_·7H_2_O 0.29 g/L, H_3_BO_4_ 2.47 g/L, CuSO_4_·5H_2_O 0.25 g/L, and MnCl_2_·4H_2_O 1.58 g/L. Meanwhile, the medium contained 34 mg/mL Cm and 100 mg/mL Amp. The engineered* E. coli* strain YJM21 [21] was inoculated to the culture broth and incubated in a gyratory shaker incubator at 37°C and 180 rpm. When OD_600_ reached 0.6, IPTG was added in final concentration of 0.5 mM, and the culture was further incubated at 30°C for 24 h.

### 2.7. Analytical Methods

Bacterial growth conditions were estimated from the optical density (OD) of the medium with a spectrophotometer (UV2310II, Shanghai Precision & Scientific Instrument Co., Ltd., China) at a wavelength of 600 nm. The concentration of isoprene was analyzed by a gas chromatograph (GC) equipped with a flame ionization detector (FID) and a TM-WAX column (25 m × 0.25 mm × 0.25 *μ*m). N_2_ was used as carrier gas. The initial column temperature was 50°C for 1 min and was increased at a rate of 6°C/min to a final temperature of 80°C, while the injector temperature was 140°C and the detector temperature was 230°C, respectively.

To identify bacterial isoprene production, peak retention times and mass spectra were compared with those of the standard. Concentrations of isoprene produced by bacterial cells were calculated by converting GC peak area to mg of isoprene via a calibration curve. Isoprene standard (TCI-EP, Tokyo, Japan) of various concentrations was added to 600 mL fermentation medium to construct a calibration curve.

## 3. Results and Discussion

### 3.1. Chemical Composition and Monosugar Composition Rate of Peanut Hull

**Figure 2:**
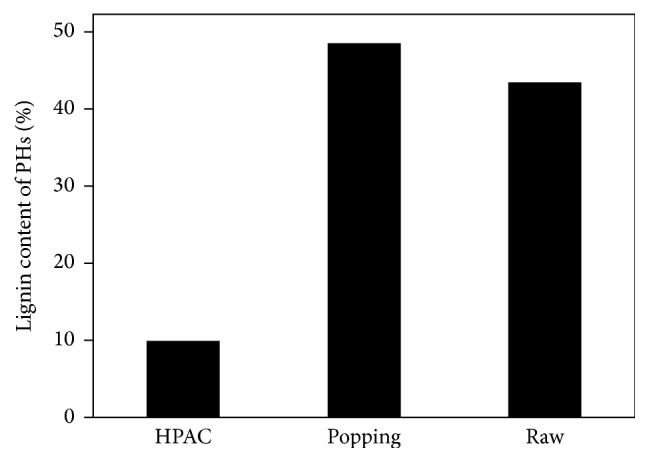
Lignin content of raw, popping, and HPAC pretreated PH.

In China, peanut hull, considered as the agricultural waste, was redundant and was not utilized very well. Meanwhile, its high polysaccharide content makes it a suitable feedstock for the production of bio-based chemicals or biofuels including isoprene. Figure 1 enumerates the chemical compositions of the peanut hull, such as ash, organic solvent extractives (OSE), holocellulose (glucose, xylose, arabinose, galactose, rhamnose, and mannose), and Klason lignin. The PH content contained 46.8% holocellulose, 5.8% ash, 4.0% OSE, and 43.4% Klason lignin. Although the lignin content of peanut hull was high and peanut hull also had significant amount of available sugars for bioconversion, this result still indicated that the lignin of PH sample needed to be removed via pretreatment process to enhance the enzymatic hydrolysis efficiency. Thus, two pretreatment methods (popping and HPAC) were conducted and the efficiency of lignin removed was evaluated.  Figure 2 had shown the difference of lignin content between raw PH and pretreated PH. Compared with raw PH, the lignin content of PH pretreated by HPAC was reduced about 77.3%, while the lignin content of PH pretreated by popping was similar to raw PH. What is more, the recovery dry mass yield of each of the pretreated samples (popping and HPAC) was approximately 75.9% and 49.3%, respectively.

**Figure 3:**
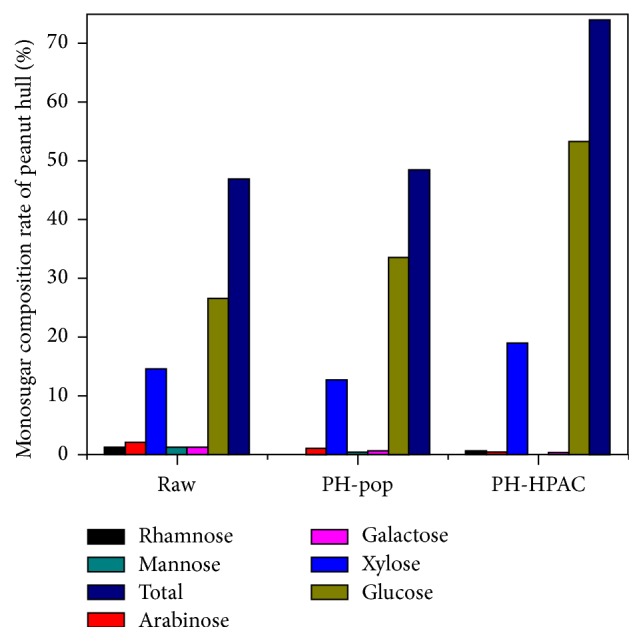
The monosugar contents of different pretreated PHs were determined using GC.

The monosugar contents of different pretreated PHs were determined using GC (Figure 3). PH was mainly composed of 46.8% carbohydrates. In these carbohydrates, xylose and glucose were major components of sugar in raw PH, comprising approximately 14.5% and 26.6% of dry mass, respectively. After pretreatment, in comparison with raw PH, total carbohydrates of popping and HPAC PH were relatively increased about 1.6% and 27.3%, respectively. Some monosugar content was relatively reduced when compared with raw PH, but glucose content was significantly increased approximately 2 times in HPAC PH. Sugar (glucose) yield of each of the samples (popping and HPAC) was approximately 95.9% and 98.8%, respectively. It was safe to reach a conclusion that the HPAC pretreatment was more effective than the popping pretreatment to increase the sugar content in the hydrolysate.

### 3.2. Surface Morphology of Pretreated Peanut Hull

**Figure 4:**
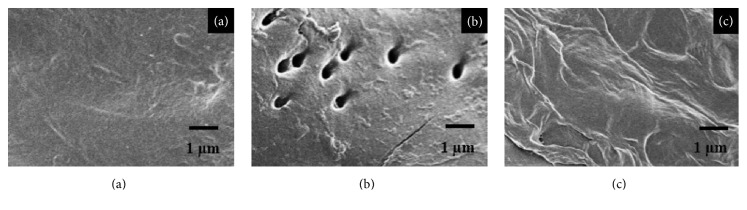
SEM images for raw (a), popping (b), and HPAC (c) pretreated PH.

The hydrolysis efficiency is directly related to the contact between cellulase and cellulose. To investigate the PH physical changes before and after pretreatment, the physical structures of them were studied with SEM (Figure 4). In comparison with the smooth and integrated surface of raw PH (Figure 4(a)), pores were present in the pretreated PH (Figure 4(b)) on account of the high temperature and releasing the high pressure quickly, so that the structure of the PH was broken down.  Figure 4(c) showed that the structure morphology of the HPAC pretreated PH was loosened and distortional, and the removed lignin might be viewed as the reason for the structure changes. No matter how destroyed the structure was or how removed the lignin was from PH, the surface area connecting cellulose and cellulose was increased. Consequently, the enzymatic hydrolysis efficiency might be increased dramatically with popping pretreatment and HPAC pretreatment.

### 3.3. Effect of Different Pretreated Methods of Peanut Hull on Isoprene Production

In our previous studies [4, 21, 22], we have engineered* E. coli* strains to biosynthesize isoprene using native MEP and exogenous MVA pathway. Compared with other studies, we found that the engineered* E. coli* strain is more effective to produce isoprene than other engineered strains such as* Cyanobacterium Synechocystis* [2],* Bacillus subtilis* [23], and* Saccharomyces cerevisiae* [24]. Hence, in this paper, we also choose the* E. coli* strain to evaluate the enzymatic hydrolysates of pretreated peanut hull.

**Figure 5:**
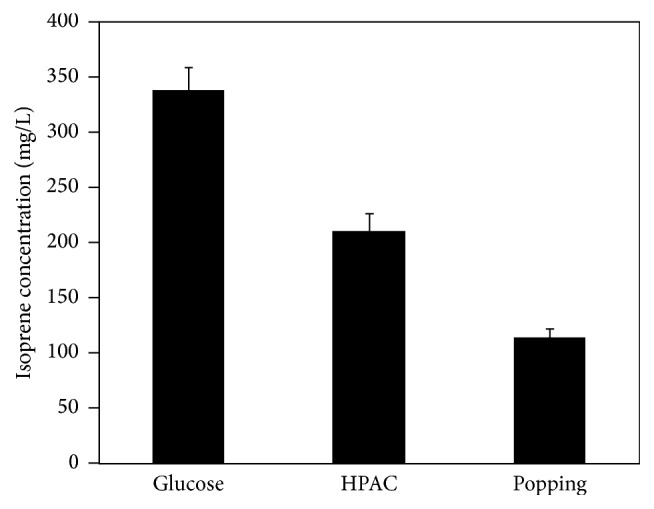
Effect of carbon sources on isoprene production. The engineered strain cultured in three different carbon sources including pure glucose, HPAC, and popping enzymatic hydrolysates. The cells were induced when OD_600_ reached about 0.6. The experiment was performed in triplicate.

Two pretreatment methods, popping and HPAC, were found to be able to destroy the lignocellulosic structure, which accordingly enhanced hydrolysis efficiency by cellulase. To ascertain the extent to which isoprene production could be influenced by these two pretreatment methods, the engineered strain was cultured in a fermentation medium with three kinds of carbon sources including pure glucose and two types of enzymatic hydrolysates using HPAC pretreatment and popping pretreatment, respectively. As was shown in Figure 5, the titer of isoprene produced by pure glucose (HPAC pretreatment and popping pretreatment) reached 338.6 mg/L, 211 mg/L, and 113.7 mg/L, respectively. The results demonstrated that the isoprene production from pure glucose fermentation was about 1.6 times and 3 times higher than that from HPAC and popping pretreatments, respectively, and using HPAC pretreatment cultures could produce approximately 1.9 times more isoprene production compared to using the popping pretreatment, with all the other conditions being the same.

Since lignocellulosic feedstocks mainly consist of cellulose, hemicellulose, and lignin [25], inhibitors exist in raw material and would be released during the course of pretreatment [26]. In addition, the concentration of inhibitors relied on the types of lignocellulosic feedstock and the different pretreatment methods utilized [26, 27]. Consequently, in this work, the reason leading to the significant difference in isoprene production among the three kinds of carbon sources would lie in the fact that inhibitors existed in two types of hydrolysates obtained by the HPAC and popping methods.

In this paper, to determine the categories and concentrations of inhibitors which were formed by HPAC and popping methods, two kinds of enzymatic hydrolysates were detected using HPLC method.  Table 1 showed that there are four kinds of inhibitors (formic acid, acetic acid, hydroxymethylfurfural (HMF), and furfural) in the enzymatic hydrolysates with popping pretreatment, while only three kinds of inhibitors (acetic acid, furfural, and HMF) were measured in the enzymatic hydrolysate with HPAC pretreatment.  Table 1 also notably indicated that the concentrations of acetic acid were approximated in two kinds of fermentation medium.

Different inhibitors have different detrimental effect on the cell growth and accordingly result in decrease in the yield of target product. Weak acid, such as formic acid and acetic acid, could cross the cell membrane, which resulted in the lower cell pH than normal and consequently inhibited cell growth [28, 29]. Mills et al. had also reported that formic acid had higher toxicity to* E. coli* than acetic acid [30]. Like furfural, HMF had a detrimental effect on DNA and would lead to single-strand breaks [29, 30]. Meanwhile, Martinez et al. had proven that the minimal inhibitory concentrations of furfural and HMF would attain 3.5 mg/mL and 4.0 mg/mL, respectively [31]. Thus, although the concentrations of HMF and furfural produced by popping pretreatment were higher than those of HPAC pretreatment, they were still too low to produce a significant impact on isoprene production.

Therefore, based on the above discussion, it can be concluded that formic acid in enzymatic hydrolysate of popping pretreatment would be the dominant factor for lower isoprene production. Additionally, acetic acid existed in HPAC hydrolysis which resulted in lower isoprene production by HPAC pretreatment than that by pure glucose.

### 3.4. Detoxification Effect of Enzymatic Hydrolysate of HPAC PH on Isoprene Production

To further enhance the production of isoprene, it is essential to remove inhibitors from enzymatic hydrolysate by using a proper method. Among various detoxification methods, activated charcoal claims the advantage of being cost effective and possessing higher capacity to absorb compounds, especially lignin-derived inhibitors and acetic acid, without affecting levels of sugar in hydrolysate [13, 32, 33]. In addition, it is safe and easy to be manipulated using activated carbon.

**Figure 6:**
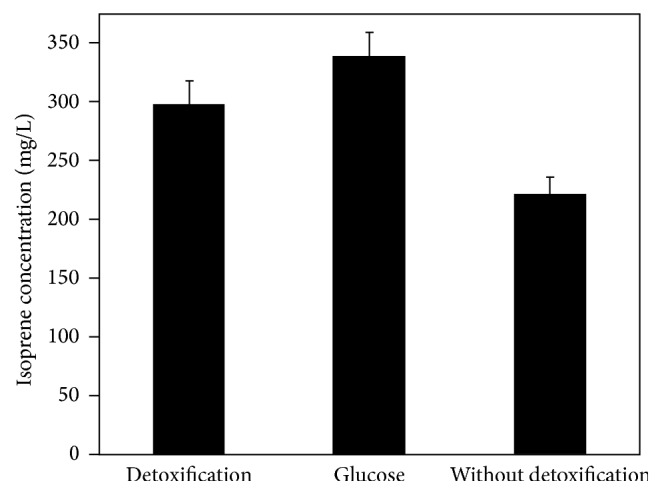
Detoxification effect of enzymatic hydrolysate of peanut hull on isoprene production. When OD_600_ reaches 0.6–0.9, cultures were induced at 30°C for 24 h using 0.5 mM IPTG. All the experiments were carried out in triplicate.

After the HPAC pretreated hydrolysate was detoxified with activated charcoal, fermentation was performed with detoxification hydrolysate and raw hydrolysate, respectively. As was seen in Figure 6, the isoprene production of detoxification of HPAC hydrolysate reached 297.5 mg/L, with an increase of up to 41% compared with raw hydrolysate without detoxification. Based on the data shown in Table 1, through detoxification with activated charcoal, the concentrations of acetic acid, HMF, and furfural were reduced by 76.90%, 72.83%, and 78.77%, respectively. The results indicated that inhibitors, especially acetic acid in enzymatic hydrolysates of peanut hull, were inhibitory to engineering* E. coli*, and the removal of inhibitors from fermentation medium consequently led to a remarkable increase in the isoprene production.

### 3.5. The Difference in Gas Composition and Concentration between Glucose and Enzymatic Hydrolysis

**Figure 7:**
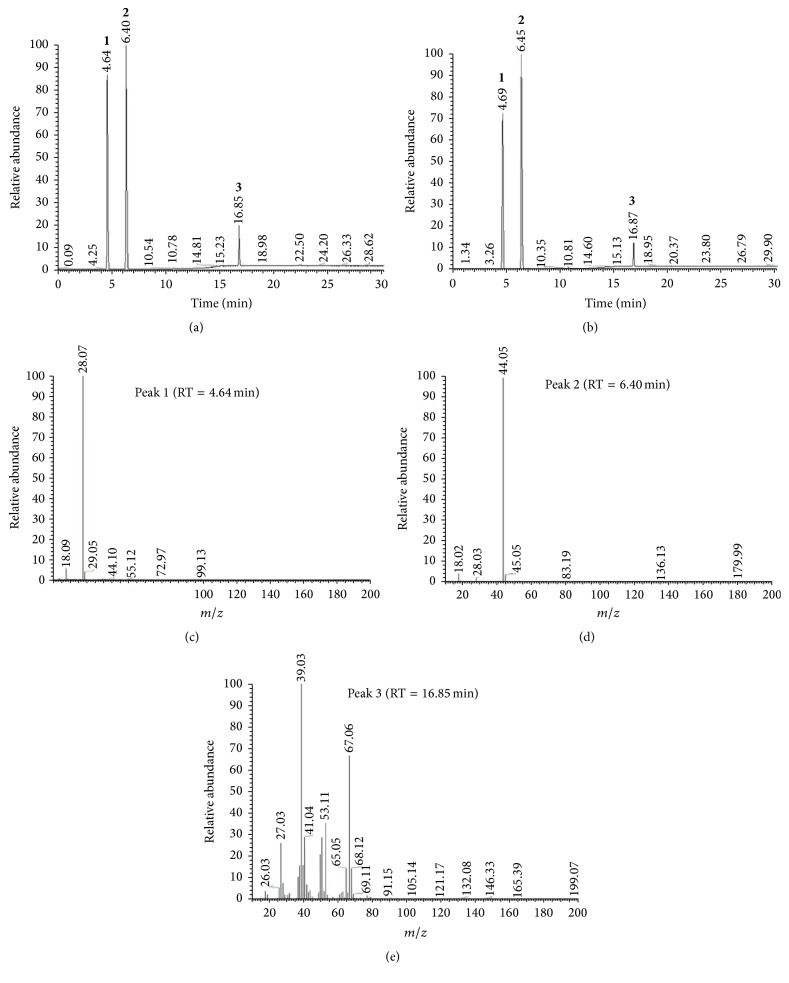
GC-MS analysis of fermentation gases. (a) GC map of gases composition of fermentation on glucose; (b) GC map of gases composition of fermentation on HPAC enzymatic hydrolysate; (c) MS map of peak 1 (N_2_); (d) MS map of peak 2 (CO_2_); (e) MS map of peak 3 (isoprene).

To detect the variation of gas composition by different carbon sources during fermentation, the engineered strain YJM21 was cultured in the fermentation medium containing pure glucose or enzymatic hydrolysate with HPAC pretreatment as the carbon source. As shown in Figure 7 and Table 2, the gas composition produced by pure glucose or enzymatic hydrolysis of peanut shell remained the same, while the gas concentration turned out to be different. The gas composition consisted of N_2_, isoprene, CO_2_, and a small amount of unknown gas. N_2_, CO_2_, isoprene, and the unknown gas which were produced by pure glucose accounted for 42.13%, 48.66%, 8.8%, and 0.41% of all gases, respectively, while those gases produced by enzymatic hydrolysate accounted for 39.34%, 54.41%, 5.96%, and 0.29%, respectively. The results indicated that though the fermentation medium constituted by enzymatic hydrolysate of peanut shell had no impact on the categories of gas composition, it could reduce the concentration of isoprene and increase the content of CO_2_ in the whole fermented gases.

## 4. Conclusions

Peanut hull proves to be a promising feedstock to produce bio-based chemicals and biofuels due to its easy availability and polysaccharide content characteristics. In this study, the use of peanut hull for isoprene production was explored. Two pretreatment methods, HPAC and popping, were carried out prior to enzymatic hydrolysis, which could destroy the lignocellulosic biomass structure. The isoprene production using HPAC pretreatment was found to be about 1.9-fold higher than that using popping pretreatment. To further increase the isoprene yield, the enzymatic hydrolysate of HPAC was detoxified by activated carbon. The engineered strain YJM21 fermented on the detoxified enzymatic hydrolysate could accumulate 297.5 mg/L isoprene, which accounted for about 90% of isoprene production by YJM21 fermented on pure glucose (338.6 mg/L). This work is considered to be the first attempt to produce isoprene by* E. coli* using peanut hull as the feedstock, and it also provides evidence that another lignocellulose material, peanut hull, could be regarded as a promising feedstock for bio-based chemicals or biofuels of industrial production.

## Figures and Tables

**Table 1:** The types and concentrations of inhibitors in different fermentation medium.

Concentration (mg/mL)	HPAC (detoxified hydrolysate)	HPAC (raw hydrolysate)	Popping
Formic acid	0	0	0.2445
Acetic acid	0.004	0.01775	0.0229
HMF	1.18 × 10^−5^	4.36 × 10^−5^	0.0119
Furfural	2.15 × 10^−5^	1.01 × 10^−4^	1.42 × 10^−3^

**Table 2:** Effect of different carbon sources on concentration of different fermentation gases.

Gas composition (%)	Glucose	HPAC enzymatic hydrolysate	Retention time (min)
N_2_	42.13	39.34	4.69
CO_2_	48.66	54.41	6.45
Unknown gas 1	0.24	0.19	10.78
Unknown gas 2	0.17	0.10	15.13
Isoprene	8.8	5.96	16.87
